# A Swath Label-Free Proteomics insight into the Faah^−/−^ Mouse Liver

**DOI:** 10.1038/s41598-018-30553-z

**Published:** 2018-08-14

**Authors:** Zeeshan Hamid, Maria Summa, Andrea Armirotti

**Affiliations:** 1 D3Validation, Fondazione Istituto Italiano di Tecnologia, via Morego 30, 16163 Genova, Italy; 2 Scuola Superiore Sant’Anna. via Piazza Martiri della Libertà, 33, 56127 Pisa, Italy; 3 Analytical Chemistry and In-vivo Facility, Fondazione Istituto Italiano di Tecnologia, via Morego 30, 16163 Genova, Italy

## Abstract

Fatty acid amide hydrolase (FAAH) is an important enzyme for lipid metabolism and an interesting pharmacological target, given its role in anandamide breakdown. The FAAH^−/−^ genotype is the most widely used mouse model to investigate the effects of a complete pharmacological inhibition of this enzyme. In this paper, we explore, by means of label-free SWATH proteomics, the changes in protein expression occurring in the liver of FAAH^−/−^ knockout (KO) mice. We identified several altered biological processes and pathways, like fatty acid synthesis and glycolysis, which explain the observed phenotype of this mouse. We also observed the alteration of other proteins, like carboxylesterases and S-methyltransferases, apparently not immediately related to FAAH, but known to have important biological roles. Our study, reporting more than 3000 quantified proteins, offers an in-depth analysis of the liver proteome of this model.

## Introduction

Fatty acid amide hydrolase (FAAH) is a serine hydrolase that cleaves bioactive endocannabinoids (ECs)^1^ including anandamide (AEA, N-arachidonoylethanolamide), a lipid that has been shown to be involved in a number of biological functions and pathologies, including several neurodegenerative disorders like multiple sclerosis^2^ and Parkinson’s disease^3^. AEA, besides its role in neural generation of pleasure and motivation^4^, has also been shown to inhibit human breast cancer cell proliferation and implantation of embryo in early stages of development^5–7^. Given the role of AEA, FAAH has been extensively studied and investigated as potential pharmacological target, and several inhibitors for this enzyme have been developed in the last years^8,9^. Very recently, though (2016), a dramatic failure of a clinical trial for safety of a FAAH inhibitor has put a serious question mark on FAAH pharmacology, although subsequent work demonstrated very relevant off-target liabilities^10^ of the inhibitor used. In FAAH pharmacology, the most widely used model to investigate the effects of total absence of FAAH activity is the FAAH^−/−^ genotype^11^. FAAH^−/−^ mice show several well-characterized phenotypes. Due to their impaired ability to hydrolyze AEA, these mice show altered anxiety^12^ and other emotional^13^ behaviors and show reduced response to pain^14^ and inflammatory^15^ stimuli. In addition to the role of FAAH in neural health, a number of studies showed its effects on central metabolism with relevant effects on food intake and body weight^16^. FAAH^−/−^ mice have similar food intake when fed with normal fat diet, but they consume more high-fat food during the day compared to wild-type. Furthermore, the body weight of FAAH^−/−^ is higher and increases more with age compared to wild-type animals^16^, making these animals prone to obesity. These animals also have higher fatty acids and triacylglycerol levels in plasma and liver^16^. It is thus clear that the absence of FAAH produces a number of changes in the mouse organism. Very surprisingly, in the extensive field of FAAH biology, we could not find any paper reporting comprehensive studies of the global alterations occurring at proteome level by genetic abolition of this enzyme. All papers reporting proteomic data on this subject are about chemical proteomics, where chemical probes are used to selectively extract and identify other serine hydrolases^17,18^. Furthermore, these papers focus on brain, because of the importance of AEA in this tissue. Intrigued by the reported changes in body weight and food intake behavior^16^, we decided to perform a proteome-scale, label free proteomics investigation of the FAAH^−/−^ liver, with the aim to investigate the role of FAAH absence. Bottom-up proteomics, aiming at evaluating the levels of expression of thousands of proteins simultaneously, combined with advanced bioinformatics tools, emerged as an extremely useful approach to understand global changes occurring in a biological system following challenges^19^, including chemical^20^, environmental^21^ and genetic^22^ ones. The aim of this study, never performed before on this model, is to understand the changes in the liver proteome following this genetic abolition and to relate the observed differences with the reported metabolic phenotype. We conducted our proteomics experiments by using the SWATH label-free approach (Sequential Window Acquisition of All Theoretical mass spectra), a technique that is currently becoming state-of-the-art in LC-MS/MS based proteomics, given its precision and accuracy in measuring protein over or under expression^23^. With this protocol, tandem mass spectra from tryptic peptides are continuously acquired over the whole scan range all along the duration of the chromatographic run. Following a software-based timescale realignment of fragments to precursors, the ion currents generated by individual peptides are measured and used to quantify the abundance of the corresponding proteins^23–25^. By using dedicated bioinformatics tools^26–28^ we performed gene enrichment and ontology studies to detect the most altered biological processes and pathways resulting from the genetic abolition of FAAH.

## Results and Discussion

**Figure 1:**
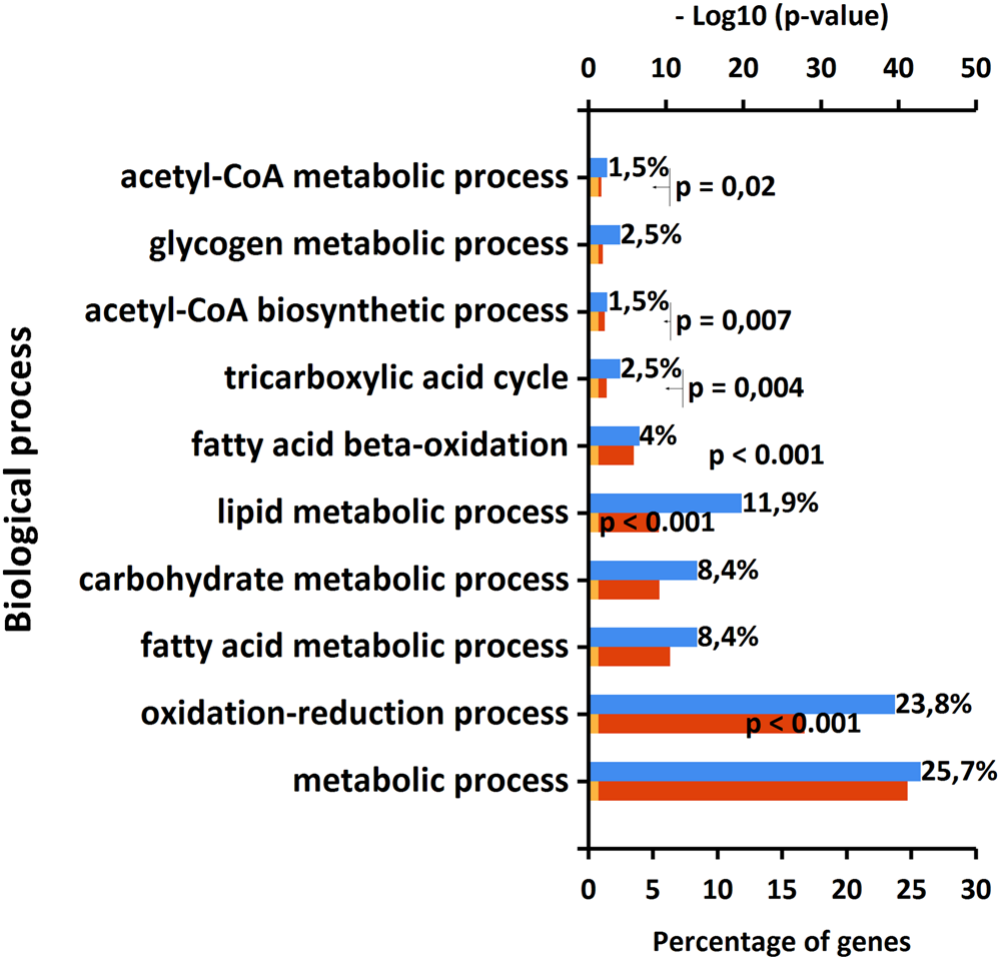
Significantly upregulated biological processes observed in the FAAH^−/−^ mouse liver.

We acquired SWATH untargeted proteomics data on liver tryptic digests from 6 FAAH^−/−^ mice and 6 wild-type. With this experiment, we quantified 3191 mouse proteins in the 12 samples. We then normalized the data by using the “Most Likely Ratio” algorithm^29^ and we performed a Principal Component Analysis (PCA) of our dataset^30^. By running a t-test, we then identified 206 proteins being significantly upregulated and 186 significantly downregulated in the KO genotype. Supplementary File 1 reports all the results of our proteomics investigation. Supplementary Data reports the full set of multivariate analysis of our proteomics data (Figs S1–S3). We also checked the levels of expression of FAAH in the animal liver and our SWATH data confirm the almost complete abolition of FAAH from the proteome of these animals (−85% fold change compared to wild-type, see Supplementary File 1 and Fig. S4). We then performed a gene enrichment analysis of our dataset by using the FUNRICH free tool^31^, on both over and under expressed protein lists. The full outcome of our enrichment analysis is reported in Supplementary File 2. We first explored the biological processes significantly upregulated by the absence of FAAH, according to the Storey-Tibshirani Q-value^32^. Figure 1 reports the biological processes upregulated in FAAH^−/−^ mice liver.

**Figure 2:**
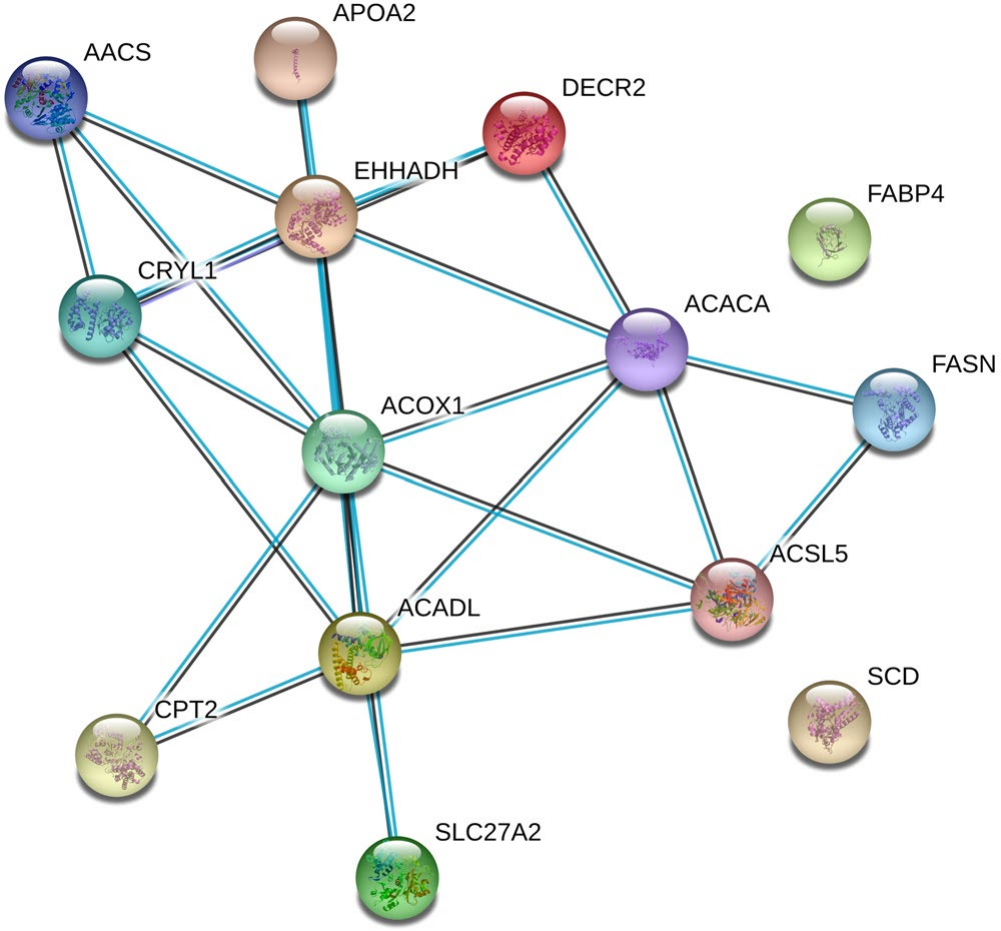
Protein association analysis performed by STRING. 13 out of 15 nodes (representing individual proteins) are associated based on co-expression data (connecting black lines) or are reported to be associated by curated databases (light blue lines).

Our data indicate that metabolic processes are strongly upregulated, with **52** overexpressed proteins. A deeper investigation of these proteins, performed by DAVID web based tool^26^ and KEGG pathway database^33^, revealed that PPAR signaling is the most activated pathway, as reported in Table S1 in the Supplementary Data. The PPAR signaling pathway, with the observed upregulated proteins indicated in red, is also reported in Supplementary Data (Fig. S5). AEA is a well-known activator of the PPAR signaling pathway^34,35^ and this upregulation is the response to the increased levels of this lipid. Intriguingly, all the pathways in Table S1 are represented by a total of 15 individual proteins. We then performed on these entries a protein association analysis by using the STRING^36^ data mining software. The results of this network analysis are represented in Fig. 2. All these proteins, except FABP4 and SCD (fatty acid binding protein 4 and stearoyl-coA desaturase), are known to be associated, either by experimentally determined co-expression or by co-association data reported in curated databases.

**Figure 3:**
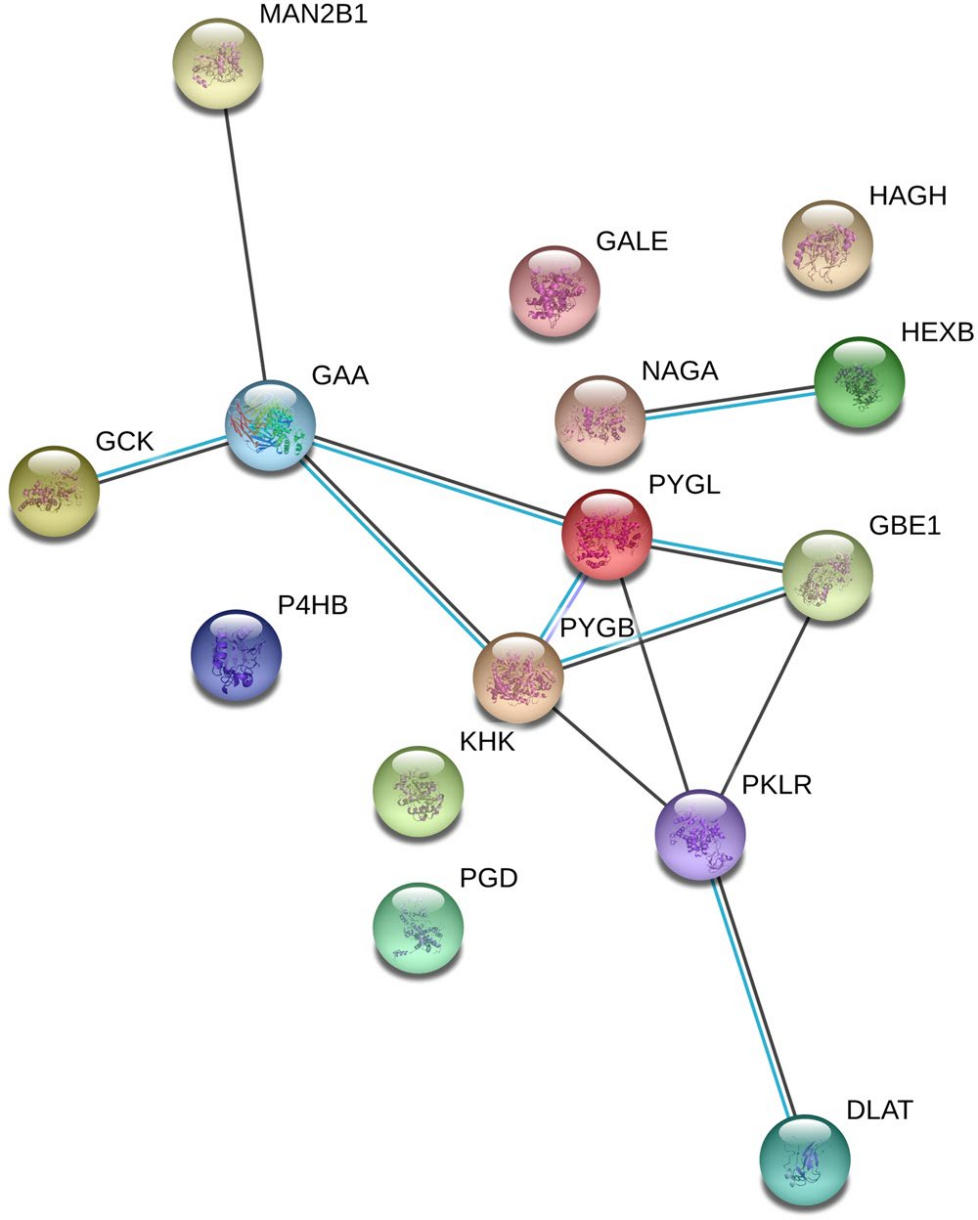
Protein association analysis performed by STRING. 8 out of 15 nodes (representing individual proteins) are associated based on co-expression data (connecting black lines).

Fatty acid binding proteins, key effectors for the intracellular transport of lipids, also show an interesting expression trend, as reported in the Supplementary Data (Fig. S6). FABP1 and FABP5 are significantly downregulated in the FAAH^−/−^ genotype, while FABP4 is upregulated. The downregulation of FABP1 and FABP5 should represent an adaptation to the excess of AEA in the tissue, while FABP4 has never been associated with AEA transport^37^. Other FABPs are present in the dataset (FABP7, FAPP_liver_ and FABP_intestinal_) but do not show any relevant alteration. In brain, FABPs are known to bind AEA^38^ and are critical for its delivery to FAAH^39^ and the inhibition of these carriers results in an analgesic phenotype^40^. Much less is known about liver. In 2016 it was demonstrated that FABP1 is the major endocannabinoid transporter in liver^41^. Our data are in agreement with this, as higher AEA levels would require lower expression of its major transporters. In this respect, our data might suggest that FABP5, another known AEA transporter^42^, might also be active in the liver. Quite surprisingly (Fig. 1 and Supplementary File 2), our gene ontology analysis also outlined the upregulation of 17 proteins related to carbohydrate metabolism (Fig. S7). We further investigated these proteins with DAVID and we identified 11 upregulated pathways, as reported in Table S2 (Supplementary Data). As for Table S1, all the pathways reported in Table S2 are represented by a total of 15 individual proteins. The corresponding STRING analysis, shown in Fig. 3, reveals that 8 of them (mannosidase, glucokinase, glucosidase, glycogen phosphorylase, glucan branching enzyme 1, pyruvate kinase and dihydrolipoamide S-acetyltransferase) are co-expressed (connecting black lines).

**Figure 4:**
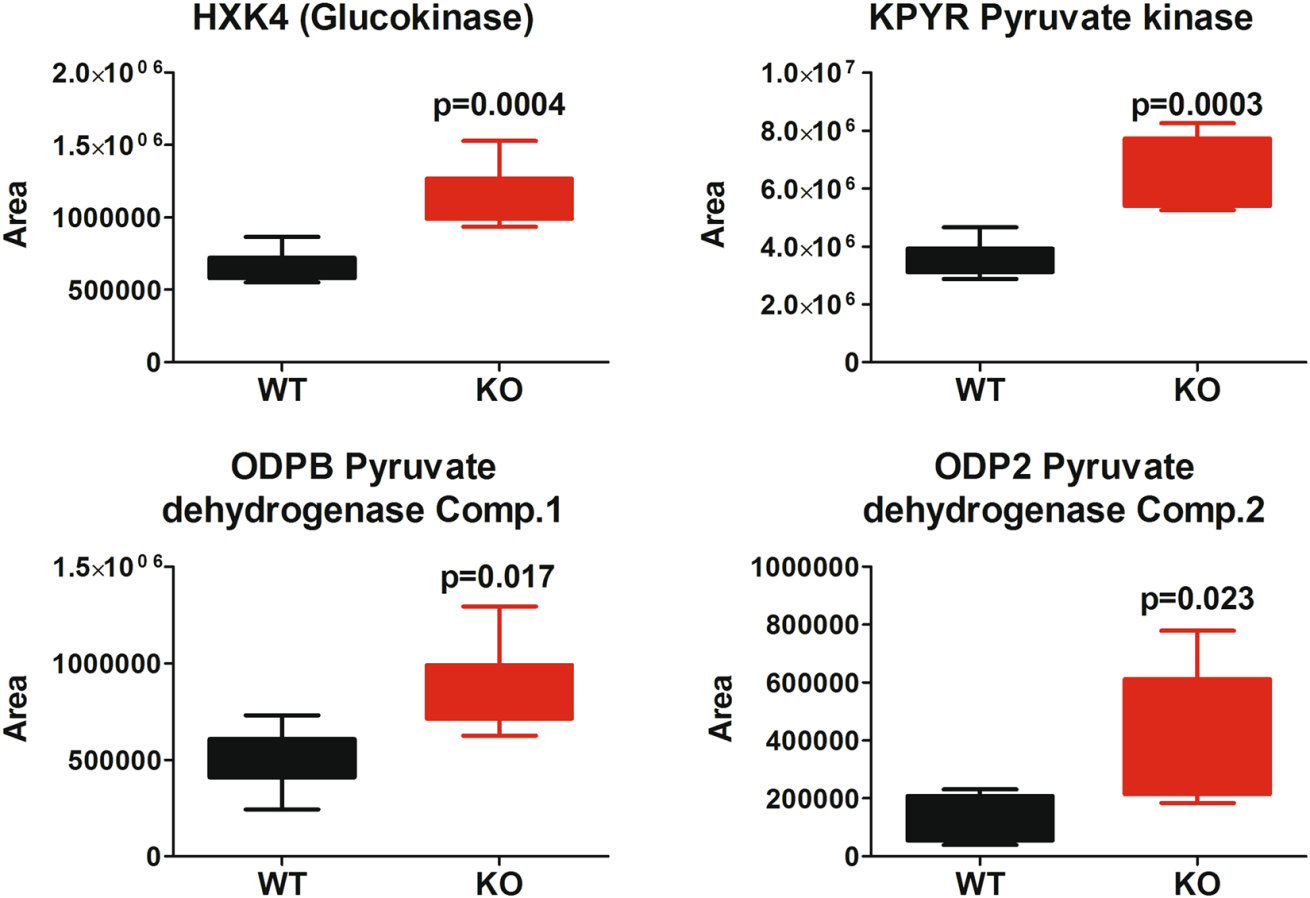
Significantly altered glucose metabolism protein observed in the dataset (statistics refers to an unpaired, two tails, Student’s t-test).

In particular, four key proteins for hexose catabolism are upregulated in our dataset (Fig. 4).

**Figure 5:**
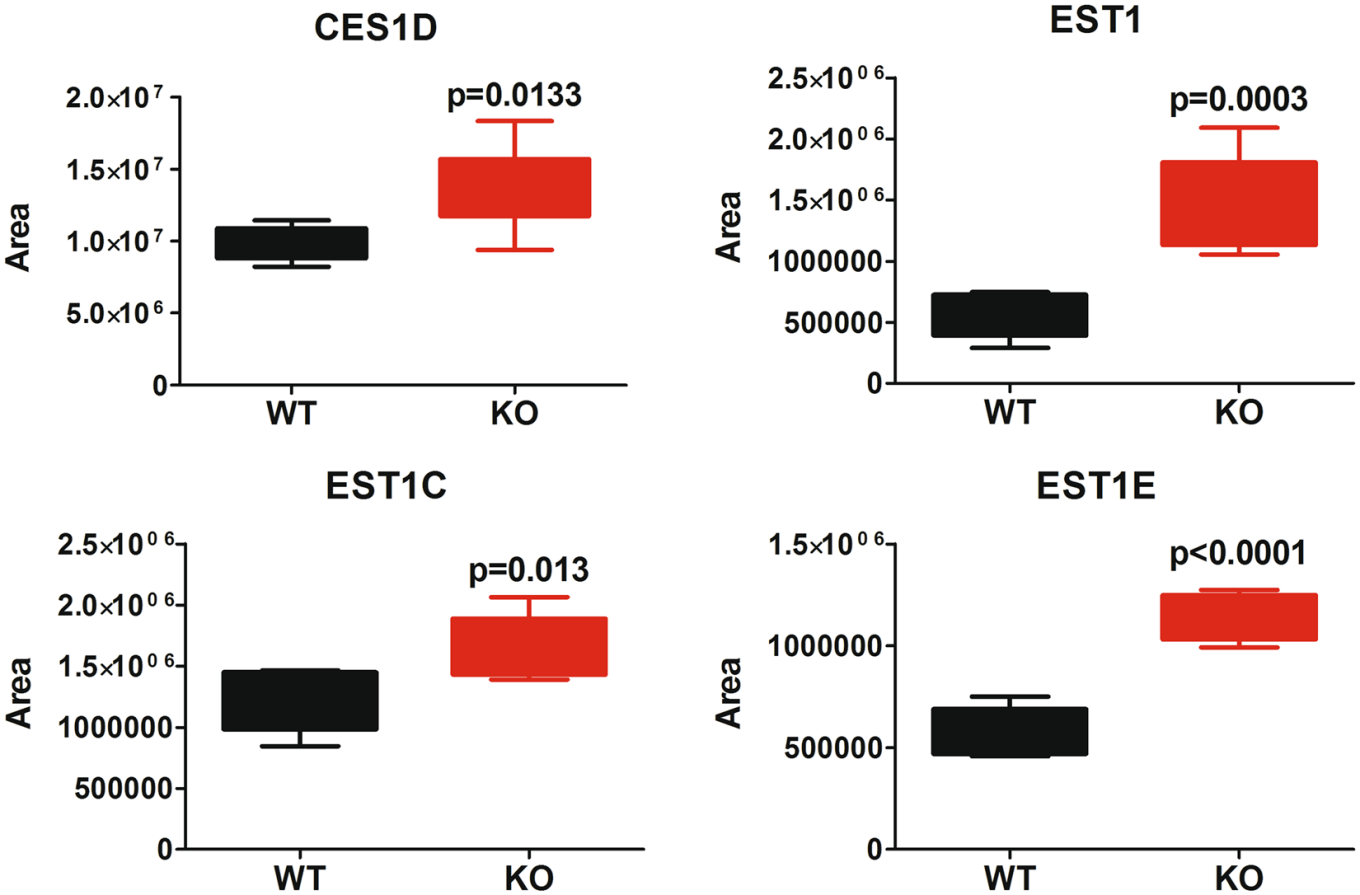
Significantly altered type1 carboxylesterases observed in the dataset (statistics refers to an unpaired, two tails, Student’s t-test).

These proteins are key effectors in the conversion of glucose into pyruvate and in the production of acetyl-CoA (by the pyruvate dehydrogenase complex). The conversion of pyruvate into acetyl-CoA occurs in the mitochondrion. Acetyl-CoA is then transported as citrate (from TCA cycle) in the cytosol (the site of fatty acid synthesis)^43^, where ATP citrate lyase (ACTLY) converts citrate back into acetyl-CoA making it available for fatty acid synthesis. ACTLY is also strongly upregulated in our dataset (+255%, p = 0.03, see Supplementary File 1). Acetyl-CoA is then used by the FAAH^−/−^ liver to sustain fatty acid synthesis, a process that our data demonstrate to be significantly upregulated (Fig. 1). The acetyl-CoA acetyltransferase, that carries the acetyl-CoA into the fatty acid synthetic process, is also upregulated (p = 0.04, THIL). 3-ketoacyl-CoA thiolase A is significantly upregulated too in the FAAH^−/−^ genotype (p = 0.006, THIKA). Based on all these evidences, it appears that one of the most prominent effects of FAAH abolition is the increased production of fatty acids, sustained by an increased production of acetyl-CoA from glycolysis, through pyruvate. This kind of regulation of glycolysis and fatty acid synthesis is known to occur in mammals^44,45^. Our data are in agreement with the reported obese phenotype of these mice, and their tendency to accumulate fats in their tissues, including liver, even under standard diet^16^. Another intriguing data in our dataset is the unexpected and very strong downregulation of betaine-homocysteine S-methyltransferase 2 (BHMT2), whose levels are as decreased as FAAH (−85%, p < e-9, see Supplementary File 1). BHMT2 belongs to the one carbon metabolism pathway, catalyzing the synthesis of methionine^46^. BHMT2, together with the downregulated thiopurine S-adenosyl transferase (TPMT,−58%, p < 0.0002, see Supplementary File 1) is among the key players of the SAM (S-adenosyl-methionine) cycle, where SAM acts as a methyl donor able to methylate substrates like protein, lipids and DNA, thus controlling a diverse array of epigenetic pathways and cellular functions^47^. Very interestingly, the complete abolition of BHMT gene also results in an altered metabolic phenotype^48^, with increased liver weight and decreased adipose tissue, resulting in *decreased* body weight under normal feeding conditions. We are wondering if the strongly decreased expression of BHMT2, subsequent to FAAH abolition, represents a compensatory mechanism or an alternative link between FAAH and energy metabolism. Another interesting result emerging from our data is the upregulation of different forms of type 1 carboxylesterase, as reported in Fig. 5.

**Figure 6:**
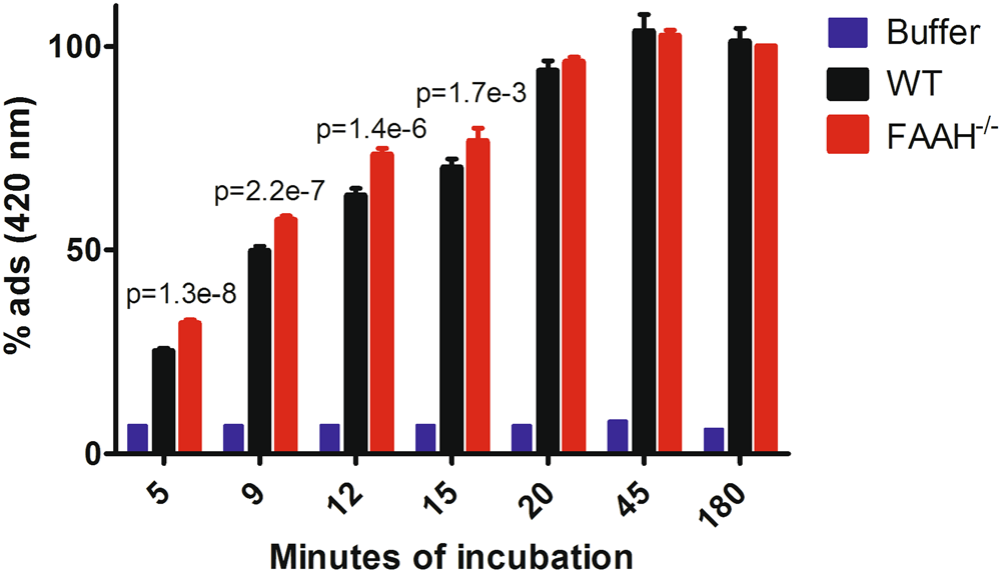
o-NPA hydrolysis to o-NP with time, catalyzed by the presence of liver homogenate (monitored by the adsorbance at 420 nm). o-NPA was dissolved at 3 mM concentration in HEPES buffer (50 mM, pH 7.4). 20 ng of liver homogenate were added to each well. Buffer refers to an incubation with the buffer only. Statistics refers to an unpaired, two tails, Student’s t-test. Mean values ± standard deviation are reported. N = 6 per group.

As an activity of carboxylesterases also toward lipid amides is reported^49,50^, the upregulation of these proteins might indicate an adaptive compensation to the absence of FAAH. Intriguingly, these enzymes are the most important players in the catabolism of cocaine and other drugs^51^. FAAH, on the other hand, has been investigated as a potential target to treat drug addiction, given the rewarding effects of endocannabinoids^52^ and its blockade has been associated to an higher sensitization to cocaine^53^. Since, to the best of our knowledge, the concomitant upregulation of carboxylesterases in liver has never been reported before for the FAAH^−/−^ model, this information might potentially be useful for the future perspectives of pharmacology to treat cocaine addiction. In order to translate this finding into a functional evidence, we exposed the same liver homogenates we used for proteomics to ortho-nitrophenylacetate (o-NPA) and we monitored the formation of ortho-nitrophenol (o-NP); o-NPA is the standard substrate for carboxylesterase functional assays^54^. The reaction is fast, even at room temperature and, in our conditions, it reaches a plateau in roughly 30 minutes. As reported in Fig. 6, the FAAH^−/−^ liver is *significantly faster* at hydrolyzing o-NPA compared to the WT, thus validating our proteomics data and supporting our hypothesis of an increased ability of the KO liver to metabolize other exogenous substrates bearing carboxylic ester moieties (like cocaine).

## Conclusions

In the present paper, we undertook a systematic investigation of the FAAH^−/−^ liver proteome, by performing an untargeted SWATH label-free protein expression analysis. Quite surprisingly, although this model is the most widely used in endocannabinoids pharmacology to simulate a total absence of FAAH activity, no such proteomics studies were ever reported in literature. Furthermore, to the best of our knowledge, this is the first time that *liver* is at the center of a systematic investigation of this genotype. Our analysis revealed the upregulation of proteins active in several biological processes related to fatty acid and glucose metabolism. We observed the upregulation of the PPAR signaling pathway, whose activation by anandamide and other endocannabinoids is known^35,55^. We also observed the strong upregulation of key proteins for fatty acid synthesis, like fatty acid synthase, and proteins acting as lipid transporters, including some known to bind AEA (Fig. S6). Our data also demonstrate that several key proteins for the pyruvate conversion into acetyl-CoA are upregulated (Fig. 4) to sustain fatty acid synthesis. These data are in agreement with the reported data on the FAAH^−/−^ phenotype, that is known to be prone to develop obesity through lipid accumulation^16^. We also observed a strong decrease in the expression of S-methyl transferases (BHMT2 and TPMT) known to participate to the metabolism of S-adenosylmethionine, a relevant player in epigenetic control of several biological pathways. The under-expression of BHMT2 is so strong that we are wondering if at least a part of the FAAH^−/−^ phenotype (the accumulation of fatty acids in liver, mostly) might be caused by the concomitant almost total abolition of this enzyme. Future experiments should aim at better deciphering this question, perhaps by investigating the phenotype of a double FAAH *and* BHMT2 knockout animal. Our findings on the overexpression of key metabolic enzymes might also be useful for clinical research, as FAAH polymorphisms are associated with increased obesity in human subjects^56,57^. We also observed the upregulation of type1 carboxylesterases and this data might be important for the potential of FAAH pharmacology to treat cocaine addiction. These findings, generated by an extensive shotgun proteomics investigation, were never reported before. The primary aim of our proteomic research is to share our data with the worldwide community of scientists working on FAAH and endocannabinoid pharmacology. We believe that further experiments can be envisaged based on our data. As an example, a comparison of the proteomics changes induced by the genetic abolition of FAAH with those induced by its prolonged pharmacological inhibition, perhaps by a chronic systemic administration of a reference inhibitor. Other experiments should also aim at elucidating the role of insulin (if any) in the observed glycolysis to fatty acid synthesis switch, a topic that has already been studied^16,56^. All our data, including raw files and quantification results are publicly available through the PRIDE proteomics data repository^58^.

## Methods

### Chemicals, Reagents And Analytical Standards

All chemicals and reagent used for sample preparation and LC-MS/MS analysis were purchased from Aldrich (Milano, Italy).

### Sample collection

*In-vivo* experiments were performed in accordance with the guidelines established by the European Communities Council Directive (Directive 2010/63/EU of 22 September 2010) and approved by the National Council on Animal Care of the Italian Ministry of Health. The same FAAH^−/−^ knockout mice previously described^59^ were used for this study. Male mice were kept under a 12-hour light/dark cycle (lights on at 8:00 am) under a controlled temperature of (21 ± 1 °C) and relative humidity of (55 ± 10%) conditions. Animals were fed with a standard diet. Six FAAH knockout and six wild type littermate control mice (12 weeks of age) were sacrificed and liver samples were collected, washed with Phosphate Buffered Saline (PBS) solution (0.01 M phosphate buffer, NaCl 0.138 M, KCl 0.0027 M, pH 7.4), weighted, frozen in liquid nitrogen and stored in −80 °C till further sample processing.

### Sample Preparation and Protein Digestion

Liver samples were weighed and homogenized in 5 ml of lysis buffer (150 mM sodium chloride, 50 mM Tris⋅HCl, pH 8.0, 0.5% sodium deoxycholate, 0.1% SDS, and 1% Triton X-100), containing a cocktail of protease inhibitors. An additional brain sample from a WT animal was homogenized and analyzed to build the ion library. Tissue homogenate was centrifuged at 10,000 g for 10 minutes. The supernatant was collected and total protein content was quantified using BCA assay. 50ug protein from each sample was used for downstream processing. In-solution digestion of the homogenized tissues was performed. Briefly: protein content was reduced with 5 mM TCEP in water, alkylated with 14 mM iodoacetamide and precipitated overnight using cold acetone (−20 °C). The resulting pellet was then resuspended in a 50 mM Tris-HCl buffer (pH 8) and digested overnight at 37 °C by using a mixture of Lys C and Trypsin (Promega) in 1:50 w/w ratio with protein, following the protocol recommended by the vendor.

### Peptide Fractionation and Building of The Ion Library

To build the qualitative ion library of MS/MS assays needed for SWATH protein quantification^24^ a preliminary DDA (data dependent acquisition) step was performed. Tryptic peptides from a mouse liver and a brain were fractionated offline (8 fractions each) with a high pH/low pH strategy^60^. This offline 2D fractionation strategy exploits the different behavior of tryptic peptides in reversed phase chromatography at high (first dimension, offline) and low pH values (second dimension: the LC-MS/MS run). In short: a total of 500 μg of digested proteins from each of the two tissues was loaded on SPE column conditioned at high pH (0.1% triethylamine TEA, pH 8) then eluted in 8 fractions by increasing acetonitrile concentrations (all 0.1% TEA). Each fraction was then collected, evaporated to dryness, reconstituted in 80 μl of 3% ACN (+0.1% formic acid) and analyzed with a 5600+ TripleToF instrument (SCIEX) coupled to a NanoAcquity LC system (Waters, Milford, MA, USA) and working in nanospray mode. DDA spectra were collected over a two hours acetonitrile gradient (3 to 45%, flow rate 300 nL/min). A Picofrit 75 μm X 250 mm column (New Objective, USA) was used for peptide separation. Peptides with charge states 2+ to 5+ and showing an intensity higher than 150 counts were selected as precursors for MS/MS acquisition. A survey spectrum (400–1250 *m/z*) was acquired for 250 ms, followed by 40 DDA MS/MS experiments (100–1500 *m/z*, 100 ms accumulation time each). The following slope and intercept values were used for the dynamic collision energy (CE) calculation for DDA experiments (as CE = (slope)*(m/z) + intercept): 0.0625 for all charge states and -3, -5, -6, -6 intercept values from 2+ to 5+ respectively. DDA Raw data were analyzed with ProteinPilot software (SCIEX) using the Paragon algorithm [27]. Spectra were searched against the reviewed Mus Musculus reference proteome downloaded as FASTA file from Uniprot (Proteome ID: UP000000589) in June 27, 2017, reporting 16966 proteins. Search was performed against both target and decoy databases to calculate a global 1% FDR. Carbamidomethylation of cysteine (CAM) was set as fixed modification. Methionine oxidation was the only allowed variable post translational modification. This search was done using the FDR calculation protocol described by Tang in 2008 [28]. From this dataset, only unmodified peptides were retained to build the ion library used for subsequent SWATH protein quantification. Peptides shared by more than one protein were excluded from the ion library. This activity resulted in a library of assays for 72031 distinct peptides useful to quantify 5935 proteins at 1% FDR.

### SWATH Analysis

SWATH DIA (data independent acquisitions) were performed by using the same gradient profile used for DDA experiments. 2 μg of total lysate were loaded on column. Precursor ion selection was done in the 400–1250 m/z range, with a variable window width strategy (7 to 50 Da). After a full range survey scan of 250 ms, 100 consecutive SWATH experiments (100–1500 m/z) were performed, each lasting 25 ms. Collision energy for each individual SWATH experiment was automatically calculated by the acquisition software based on the *m/z* window by using the following equation: CE = 0.063(*m/z*) −3.24. An increasing CE spread (5 to 10 eV from the lowest to the highest end of the scan range) was applied to ensure the optimal fragmentation of peptides. DIA raw data were analyzed by using the SWATH microapp embedded in PeakView software (SCIEX). Peptides from serum albumin were used for retention time recalibration between the runs. The following criteria were used for DIA quantification: minimum peptide confidence 90%, 50 ppm maximum mass tolerance, 15 minutes maximum RT tolerance, 6 MRM transitions per peptide used for quantification, modified peptides were not allowed (and were not included in the library). The same LC gradient was used for DDA and SWATH experiments. Data were analyzed used the PeakView software (SCIEX). Multivariate data analysis and t-test statistics were performed by using Markerview software (SCIEX).

### Carboxylesterase Activity Assay

Carboxylesterase activity in the liver homogenate was performed by monitoring the conversion of o-NPA into o-NP catalyzed by this enzyme, as already described^54^. In short, substrate o-NPA 3 mM in HEPES buffer (50 mM, pH 7.4) was incubated with liver homogenate (final dilution 1:100 in volume) at 25 °C. The experiment was performed in a 96-well plate. Based on our BCA data, a total 20 ng of protein was added to each well. The formation of o-NP was monitored with a plate reader, by measuring the adsorbance at 420 nm over time.

### Gene Enrichment And Protein Association Analysis

The set of over or downregulated proteins were analyzed against the *Mus Musculus* proteome by using the FUNRICH software tool^27,31^ to detect the most altered biological processes. The corresponding Q-value^32^ was used to detect significant processes (q < 0.05). DAVID web-based tool^26^ was then used to link the list of genes for each process with the corresponding set of biological pathways browsed and explored from the KEGG database^33,61^. The full list of quantified proteins was used as background list and the lists of significantly up- or down- regulated proteins as target lists^62^. For DAVID the Benjamini-Hochberg p-value^63^ (corrected for multiple testings) was used to detect significantly altered pathways. STRING software^36^ was used to mine data on protein association networks.

## Electronic supplementary material

Supplementary Data

Supplementary File 1

Supplementary File 2

## Data Availability

All the data, including raw files and quantification results are publicly available through the PRIDE proteomics data repository with identifier: PXD010087.
