# Supplementary material for: A Swath Label-Free Proteomics insight into the Faah−/− Mouse Liver
Source: Sci Rep. 2018 Aug 14;8:12142. doi: 10.1038/s41598-018-30553-z (PMC6092373; doi:10.1038/s41598-018-30553-z)

SUPPLEMENTARY DATA

A SWATH LABEL-FREE PROTEOMIC INSIGHT INTO THE FAAH$^{-/-}$ MOUSE LIVER

Zeeshan Hamid[1, 2], Maria Summa[3] and Andrea Armirotti[3]*

1) *D3Validation, Fondazione Istituto Italiano di Tecnologia, via Morego 30, 16163 Genova*

2) Scuola Superiore Sant'Anna. via Piazza Martiri della Libertà, 33, 56127 Pisa, Italy

3) Analytical Chemistry and In-vivo Facility, Fondazione Istituto Italiano di Tecnologia, via Morego 30, 16163 Genova

*Dr. Andrea Armirotti, Istituto Italiano di Tecnologia,

via Morego 30, 16163 Genova, Italy.

andrea.armirotti@iit.it. Phone: +3901071781938

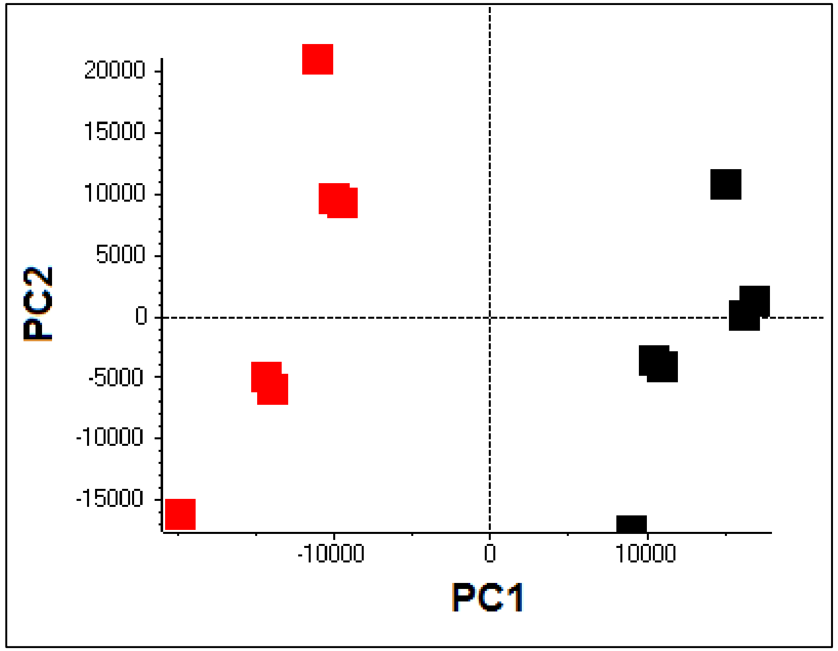

Figure S1: PCA analysis for WT (black) vs FAAH$^{-/-}$ (red) liver proteomic data. The figure reports the Scores plot for PC1 (25%) and PC2 (16%). The dataset was Pareto scaled. The plot clearly indicates that the two genotypes show significantly different protein expression profiles.

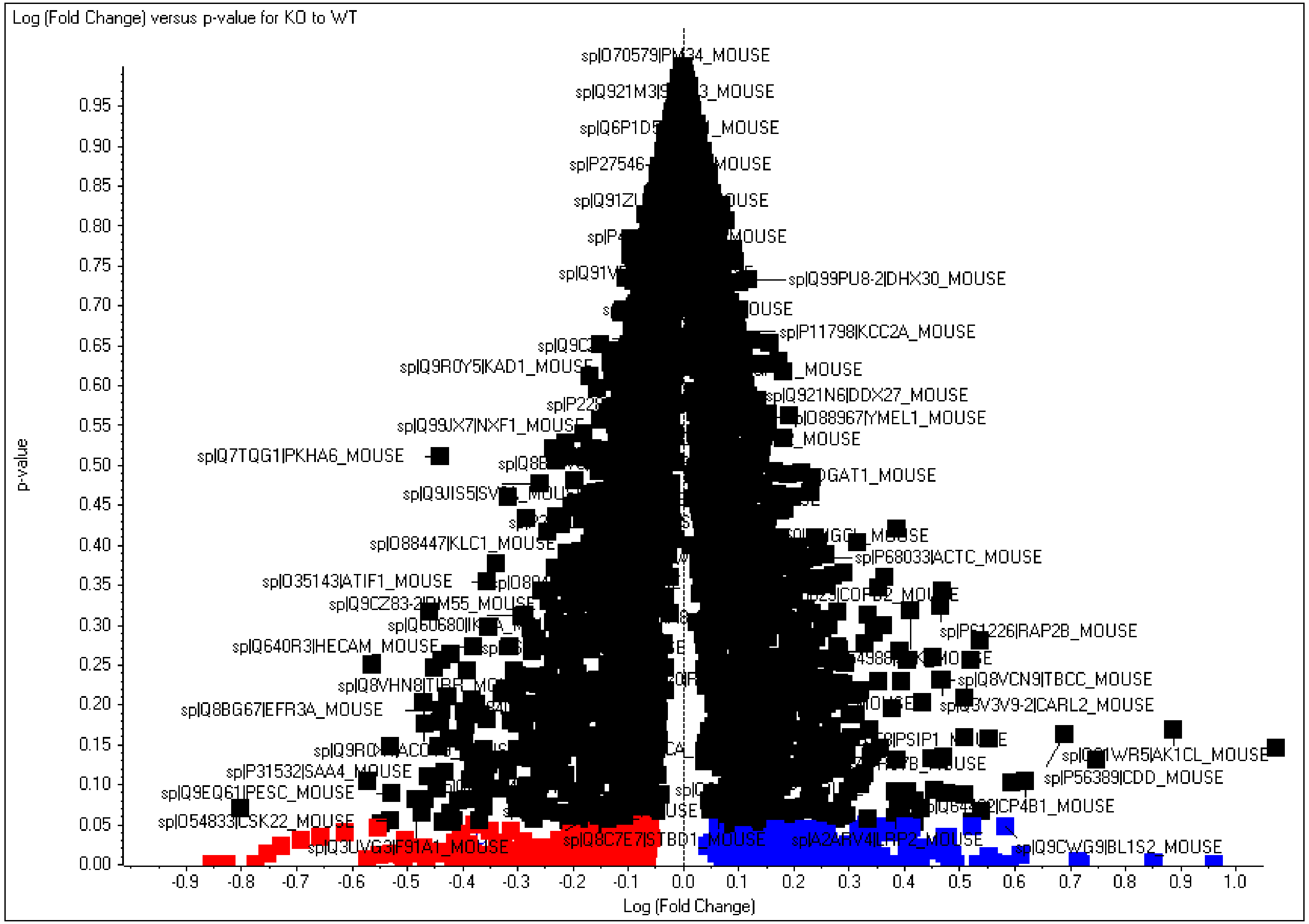

Figure S2. Volcano plot reporting the Log of the fold change values versus the corresponding p-value in the t-test comparison of WT and KO. Each dot represents a protein in the dataset. The large majority of the proteome is unaltered (black dots). Significantly pregulated proteins are in blue, downregulated in red.

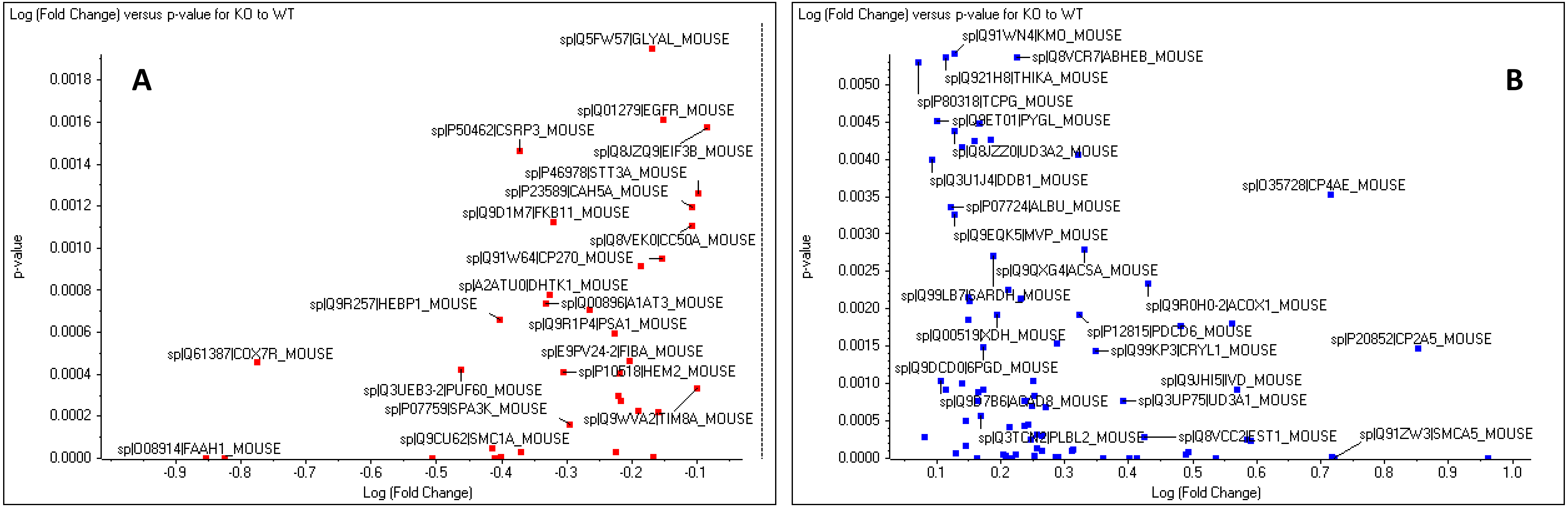

Figure S3. Bottom left (downregulated, panel A) and bottom right (upregulated, panel B) magnifications of the plot shown in Supplementary File 2. Each dot represents a protein in the dataset.

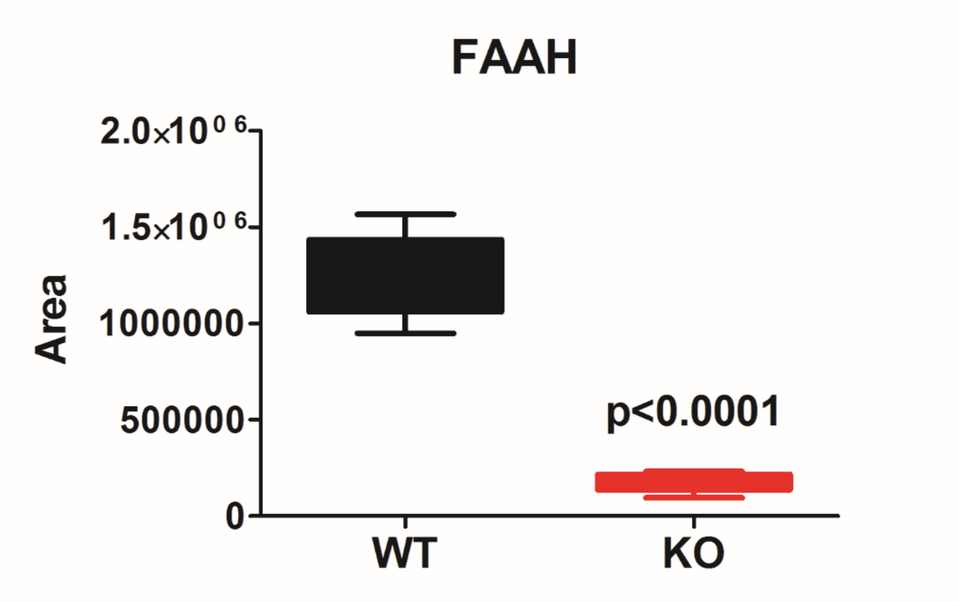

Figure S4. Expression levels of FAAH in mouse liver (WT vs KO, results of a two-tailed, unpaired t-test)

Term	Genes	N	%	Corrected p-value
PPAR signaling pathway	SCD1, ACOX1, APOA2, CPT2, EHHADH, FABP4, ACADL, ACAA1A, SLC27A2, ACSL5	10	0,4	1,1E-13
Fatty acid metabolism	SCD1, ACOX1, CPT2, EHHADH, ACACA, FASN, ACADL, ACAA1A, ACSL5	9	0,3	1,7E-13
Fatty acid degradation	ACOX1, CPT2, EHHADH, ACADL, ACAA1A, ACSL5	6	0,2	2,2E-7
Peroxisome	ACOX1, EHHADH, DECR2, ACAA1A, SLC27A2, ACSL5	6	0,2	2,4E-6
Fatty acid biosynthesis	ACACA, FASN, ACSL5	3	0,1	1,7E-3
Biosynthesis of unsaturated fatty acids	SCD1, ACOX1, ACAA1A	3	0,1	5,4E-3
Valine, leucine and isoleucine degradation	EHHADH, AACS, ACAA1A	3	0,1	1,9E-2
Metabolic pathways	ACOX1, CRYL1, EHHADH, ACACA, FASN, ACADL, ACAA1A, ACSL5	8	0,3	2,1E-2

Table S1. Biochemical pathways most significantly altered by the genetic abolition of FAAH. P-values reported in the last columns are corrected for multiple testing (Benjamini-Hochberg)

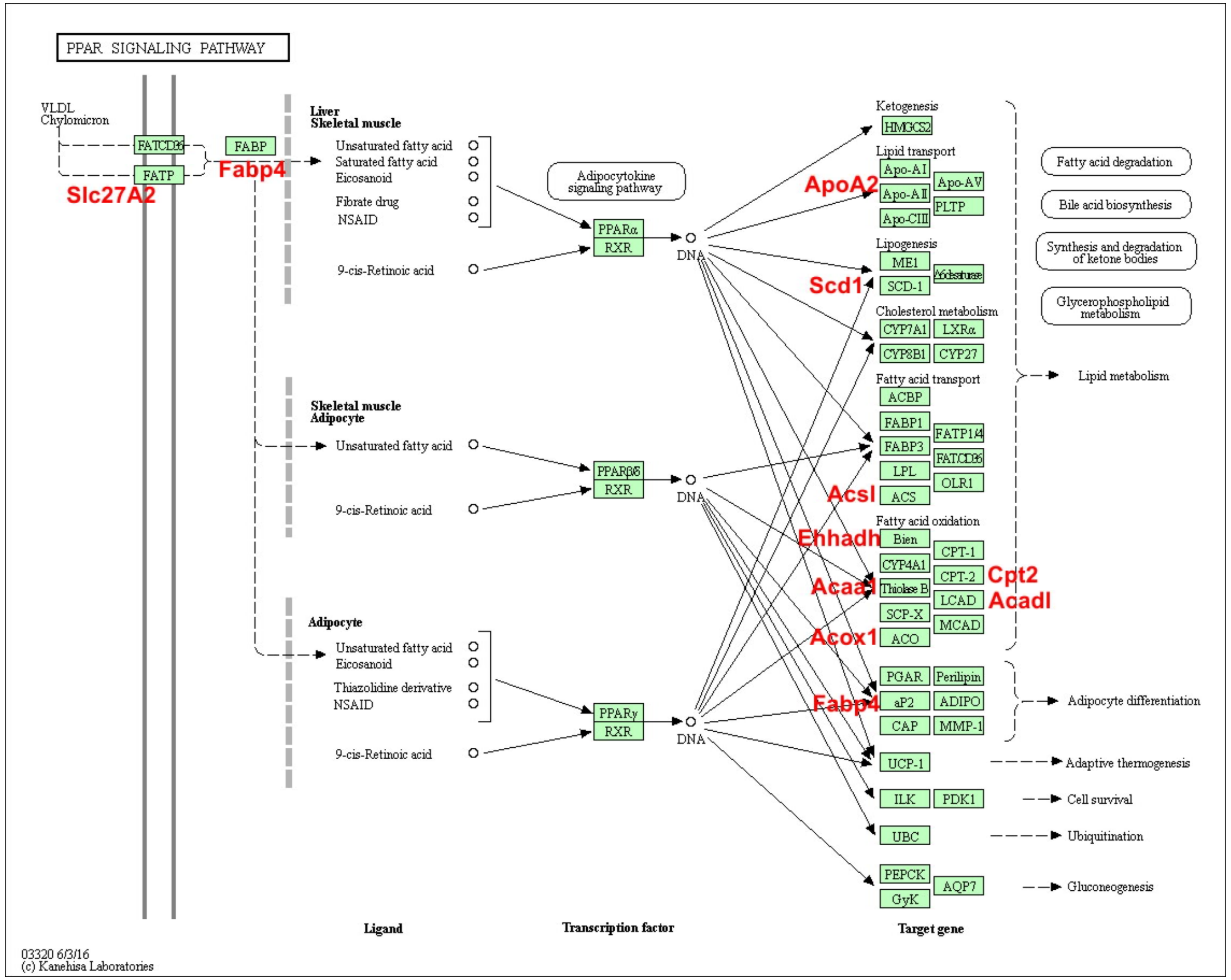

Figure S5. PPAR signaling pathway, taken from the KEGG database. The upregulated proteins observed in the FAAH$^{-/-}$ mouse liver are indicated in red. Pathway map was reproduced upon kind permission from Kanehisa Laboratories (Kanehisa, M. and Goto, S.; KEGG: Kyoto Encyclopedia of Genes and Genomes. Nucleic Acids Res. 28, 27-30 (2000).

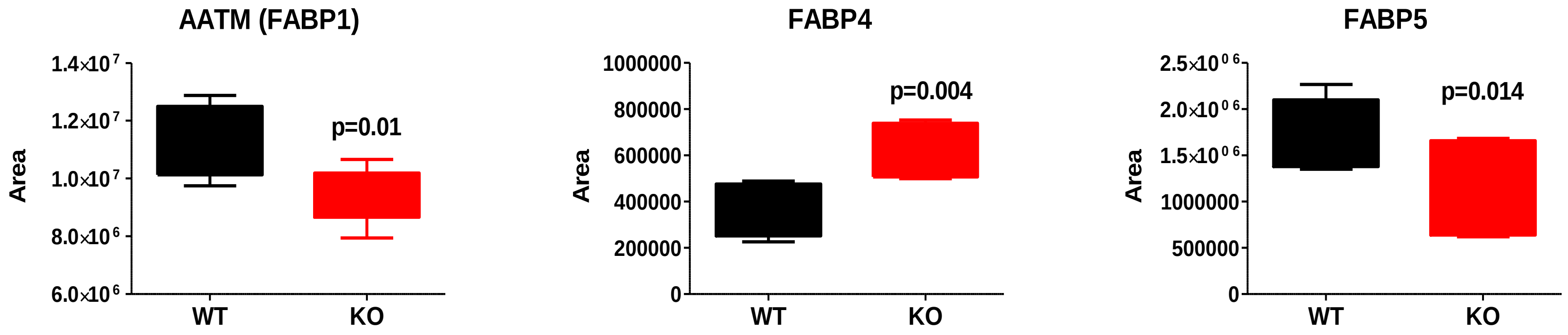

Figure S6: Significantly altered fatty acid binding proteins observed in the dataset (statistics refers to an unpaired, two tails, Student's t-test)

Term	Genes	N	%	Corrected p-value
Starch and sucrose metabolism	GBE1, GCK, PYGL, GAA, PYGB	5	0,2	7,1E-6
Metabolic pathways	KHK, GBE1, GCK, PYGL, HEXB, PGD, PKLR, GAA, GALE, DLAT, PDHB, PYGB	12	0,4	8,3E-6
Pyruvate metabolism	HAGH, PKLR, DLAT, PDHB	4	0,1	4,2E-4
Carbon metabolism	GCK, PGD, PKLR, DLAT, PDHB	5	0,2	3,2E-4
Glycolysis / Gluconeogenesis	GCK, PKLR, DLAT, PDHB	4	0,1	1,2E-3
Biosynthesis of antibiotics	GCK, PGD, PKLR, DLAT, PDHB	5	0,2	2,3E-3
Glucagon signaling pathway	GCK, PYGL, PDHB, PYGB	4	0,1	3,0E-3
Lysosome	HEXB, NAGA, GAA, MAN2B1	4	0,1	4,6E-3
Galactose metabolism	GCK, GAA, GALE	3	0,1	4,9E-3
Insulin signaling pathway	GCK, PYGL, PKLR, PYGB	4	0,1	5,5E-3
Amino sugar and nucleotide sugar metabolism	GCK, HEXB, GALE	3	0,1	9,3E-3

Table S2. Biochemical pathways for carbohydrate metabolism most significantly altered by the genetic abolition of FAAH. P-values reported in the last columns are corrected for multiple testing (Benjamini-Hochberg)

Figure S7.

Glycolysis pathway, taken from the KEGG database. The upregulated proteins observed in the FAAH$^{-/-}$ mouse liver are indicated in red. Pathway map was reproduced upon kind permission from Kanehisa Laboratories (Kanehisa, M. and Goto, S.; KEGG: Kyoto Encyclopedia of Genes and Genomes. Nucleic Acids Res. 28, 27-30 (2000).

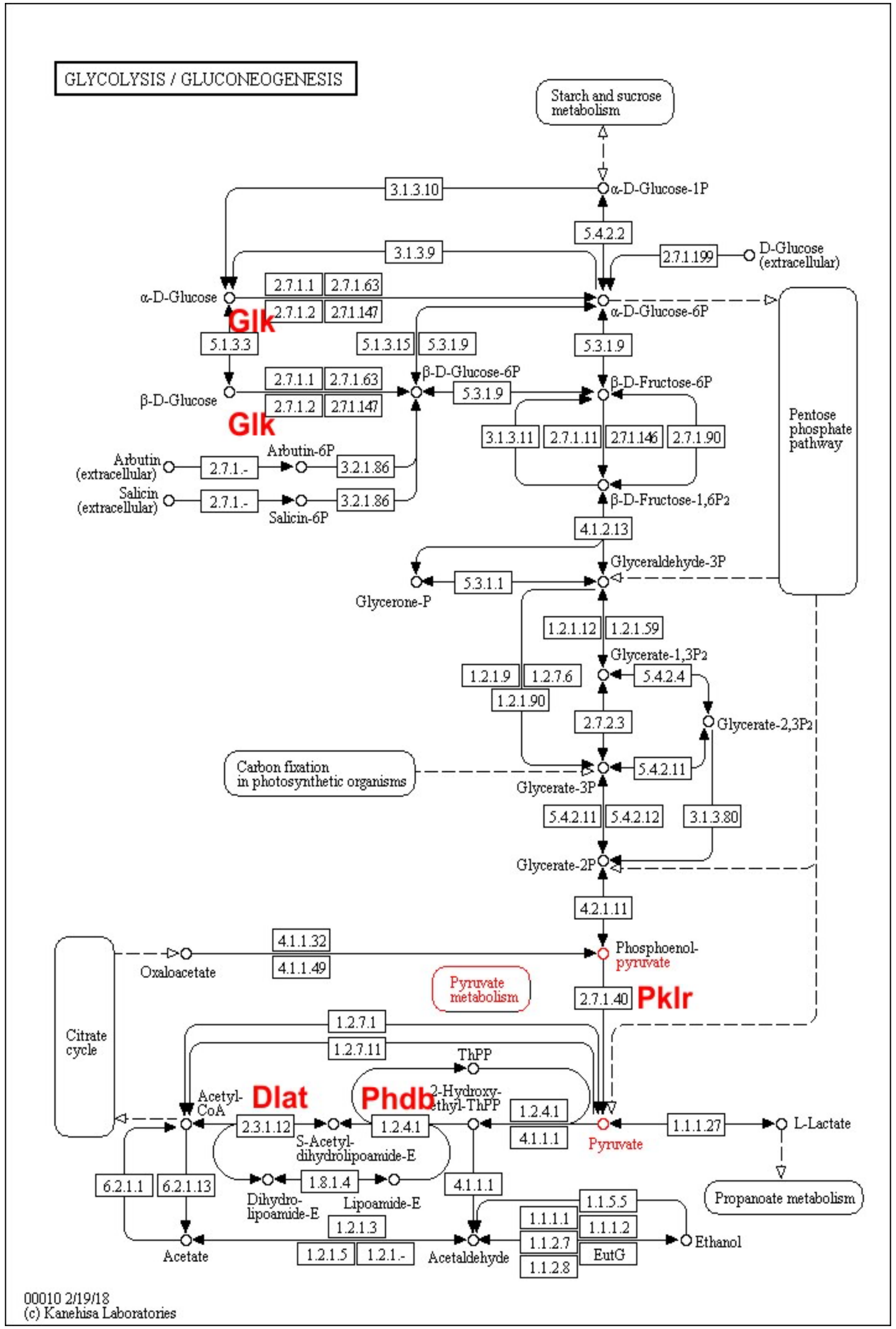

Supplement: Supplementary file 1 — Supplementary Data [file 41598_2018_30553_MOESM1_ESM.pdf]
